# Repositioning HIV protease inhibitors and nucleos(t)ide RNA polymerase inhibitors for the treatment of SARS-CoV-2 infection and COVID-19

**DOI:** 10.1007/s00228-021-03108-x

**Published:** 2021-03-04

**Authors:** Nils von Hentig

**Affiliations:** 1 Sachsenhausen Practice of General Medicine, HIV-Focus, Frankfurt am Main, Germany; 2 HIVCENTER, Internal Medicine II, Goethe University Hospital, Frankfurt am Main, Germany; 3 Institute of Clinical Pharmacology, Goethe University, Frankfurt am Main, Germany; 4 Section Pharmacology, German AIDS Society, Frankfurt am Main, Germany; 5 Praxis für Allgemeinmedizin / HIV-Schwerpunkt, Ziegelhüttenweg 1-3, 60598 Frankfurt am Main, Germany; 6 HIVCENTER, Johann Wolfgang Goethe Universität, Theodor-Stern-Kai 7, 60590 Frankfurt am Main, Germany

**Keywords:** Repositioning drugs, SARS-CoV-2, HIV protease inhibitors, RNA polymerase inhibitors

## Abstract

**Aims:**

SARS-CoV-2 is a single-stranded RNA virus which is part of the ß-coronavirus family (like SARS 2002 and MERS 2012). The high prevalence of hospitalization and mortality, in addition to the lack of vaccines and therapeutics, forces scientists and clinicians around the world to evaluate new therapeutic options. One strategy is the repositioning of already known drugs, which were approved drugs for other indications.

**Subject and method:**

SARS-CoV-2 entry inhibitors, RNA polymerase inhibitors, and protease inhibitors seem to be valuable targets of research. At the beginning of the pandemic, the ClinicalTrials.gov webpage listed *n*=479 clinical trials related to the antiviral treatment of SARS-CoV-2 (01.04.2020, “SARS-CoV-2,” “COVID-19,” “antivirals,” “therapy”), of which *n*=376 are still accessible online in January 2021 (10.01.2021). Taking into account further studies not listed in the CTG webpage, this narrative review appraises HIV protease inhibitors and nucleos(t)ide RNA polymerase inhibitors as promising candidates for the treatment of COVID-19.

**Results:**

Lopinavir/ritonavir, darunavir/cobicistat, remdesivir, tenofovir-disoproxilfumarate, favipriravir, and sofosbuvir are evaluated in clinical studies worldwide. Study designs show a high variability and results often are contradictory. Remdesivir is the drug, which is deployed in nearly 70% of the reviewed clinical trials, followed by lopinavir/ritonavir, favipiravir, ribavirine, and sofosbuvir.

**Discussion:**

This review discusses the pharmacological/clinical background and questions the rationale and study design of clinical trials with already approved HIV protease inhibitors and nucleos(t)ide RNA polymerase inhibitors which are repositioned during the SARS-CoV-2 pandemic worldwide. Proposals are made for future study design and drug repositioning of approved antiretroviral compounds.

## Introduction

Since November 2019, the severe acute respiratory syndrome (SARS) and the coronavirus disease 2019 (COVID-19) caused by the new coronavirus (CoV-2) is the most severe pandemic of the past 50 years.

CoV-2 is a single-stranded RNA virus which is part of the ß-coronavirus family (like SARS 2002 and MERS 2012). The high prevalence of hospitalization and mortality, in addition to the lack of vaccines and therapeutics, forces scientists and clinicians around the world to evaluate new therapeutic options. One strategy is the repositioning of already known drugs, which were approved drugs for other indications [1].

In this case, targets for research would be the receptor used by SARS to enter the cells to be infected, ACE2, the RNA polymerase or the virus protease (Mpro), needed for fusion, replication, and embudding of new viruses in the host cells. Thus, entry inhibitors, RNA polymerase inhibitors, and protease inhibitors seem to be valuable targets of research.

The ClinicalTrials.gov webpage currently (10.01.2021) lists 376 clinical trials related to the treatment of SARS-CoV-2 of which 15 are found to be completed, although this might not be the real number due to the fact that investigators often do report final study data with time delay. Taken together with a small number of further studies not listed in the CTG webpage, the current evidence of drug treatment of COVID-19 is scarce. Thus, the majority of studies is planned or currently recruiting.

This review discusses the pharmacological/clinical background and rationale for clinical trials with already approved HIV protease inhibitors and other nucleos(t)ide RNA polymerase inhibitors which are repositioned during the SARS-CoV-2 pandemic worldwide.

### Principles of treatment

Nucleoside analogs have been already studied as potential therapeutic options against SARS infection. In general, these drugs are taken as prodrugs and have to be phosphorylated in the target cells to be active false components used by RNA polymerase leading to the abruption of viral RNA replication. This principle has been used as part of antiretroviral therapies for decades [2].

A number of in silico and in vitro studies evaluated the binding of drugs to RNA polymerase and protease [3–6].

The proteinase processing polyprotein and virus maturation is called main protease (Mpro) and has already been evaluated as an approach towards SARS treatment [7, 8]. We also know from HIV treatment, that HIV-1 protease inhibitors are highly able to deactivate Mpro by inactivating the active center of the protease being nominated as a potential drug against SARS infection [9].

Therefore, studies have been designed to evaluate the molecular interaction of HIV-1 protease inhibitors with Mpro, which are discussed later on.

### Nukleos(t)ide polymerase inhibitors

Sofosbuvir, remdesivir, and tenofovir-DF (TDF) are nucleos(t)ide analogs and thus prodrugs, which are 2–3× phosphorylated in the target cells in order to become active false components used by RNA polymerase leading to the abruption of viral RNA replication. Sofosbuvir is a uridine-analog, remdesivir is an adenosine-analog and tenofovir-disoproxilfumarate is a thymidine-analog. Most of the chain-terminating drugs are nucleoside-analogs missing a functional 3’OH-group (e.g., tenofovir, aciclovir or azidothymidin). Sofosbuvir is an exemption, because it is modified at the 2’OH-group for better antiviral activity against HCV. All of these can cause a premature chain termination by steric inhibition of the viral polymerase [10, 11]. Of these, ribavirin showed the highest binding energy to RNA polymerase (7.8 ckal/mol), but is followed by remdesivir (7.6), sofosbuvir (7.5), galidesvir (7.0), and tenofovir-DF (6.9) [10].

Remdesivir was found to be active against Ebola, although the results were not as encouraging as expected [12].

Analyses of patients (*n*=66, US, EU, and China) who received remdesivir in a compassionate use program showed, that Remdesivir standard dosing can improve the pulmonary status, help clear the virus and decrease fever in patients with severe COVID-19, especially those who had an O_2_-saturation <94% and who were oxygenated [13].

Remdesivir is now tested in trials worldwide. The two biggest trials, SOLIDARITY and DISCOVERY, initiated by the WHO and the French ANSM plan to enroll thousands of patients worldwide, of which 3200 will be included in countries of the European Union under the guidance of the French National Agency for Medicines and Health Products Safety (ANSM). The SOLIDARITY trial is an open randomized adaptive controlled trial, where patients are either assigned to receive remdesivir intravenously 100 mg daily for the duration of the hospitalization and up to 10 days total course, including a loading dose of 200 mg at inclusion or hydroxychloroquine will be given orally (in the ICU in gastrointestinal tubes) with 800 mg loading dose followed by 400 mg every day for a total of 10 days, plus standard of care treatment (SOC) in each arm [14].

The manufacturer of remdesivir, Gilead Sciences Inc., also announced the initiation of two phase 3 clinical studies in March 2020 to evaluate the safety and efficacy of remdesivir in adults diagnosed with COVID-19. These randomized, open-label, multicenter studies will enroll approximately 1000 patients at medical centers primarily across Asian countries, as well as other countries globally with high numbers of diagnosed cases. The studies will assess two dosing durations of remdesivir, administered intravenously.

Recently, remdesivir has been approved by the FDA and EMA for the treatment of severely ill COVID-19 patients, being hospitalized and O_2_-ventilated. Data on remdesivir were assessed in an exceptionally short timeframe through a so-called *rolling review*, an approach used by EMA during public health emergencies to assess data as they become available. From April 30, 2020, the CHMP began assessing data on quality and manufacturing, non-clinical data, preliminary clinical data, and supporting safety data from compassionate use programs, well in advance of the submission of the marketing authorization application on the 5th of June 2020. The recommendation was mainly based on data from study NIAID-ACTT-1. Preliminary results of this study indicate that hospitalized patients with severe COVID-19 who received remdesivir had a 31% faster time to recovery than those who received placebo (*p*<0.001). Specifically, the median time to recovery was 11 days for patients treated with remdesivir compared with 15 days for those who received placebo. Results also suggested a survival benefit, with a mortality rate of 8.0% for the group receiving remdesivir versus 11.6% for the placebo group (*p*=0.059) [15].

ClinicalTrials.gov lists a total number of 10 trials with remdesivir against SARS-CoV-2 infection (01.08.2020, https://clinicaltrials.gov/ct2/results?cond=COVID-19&term=remdesivir&cntry=&state=&city=&dist=&Search=Search)

Also, two substantial reviews came to the conclusion that remdesivir is the foremost candidate for the treatment of COVID-19 patients who receive mechanical ventilation [16, 17].

In Italy, a multi-center, randomized, double-blind, placebo-controlled (1:1) clinical study to explore the efficacy and safety of favipiravir in the treatment of adult subjects with COVID-19-moderate type started in July 2020. Subjects within 10 days of COVID-19 onset will be screened and be randomized as early as possible within 24 h following screen success. It is planned to randomize 100 subjects in a 1:1 ratio. Subjects in the test group will receive supportive care recommended in the current guidelines + favipiravir, and subjects in the control group will receive supportive care recommended in the current guidelines + placebo control; the efficacy and safety of favipiravir versus the placebo in the treatment of COVID-19-moderate type will be compared [18].

A Chinese multicenter interventional trial evaluates the combination of Favipiravir combined with tocilizumab. 150 participants will be assigned to receive either the combination of favipiravir or favipiravir or tocilizumab alone, each plus SOC.

Favipiravir will be given 1600mg BID on the 1st day and 600 mg BID from day 2 to 7, p.o.

The first dose of tocilizumab is 4~8 mg/kg as i.v. infusion and the recommended dose is 400 mg. For fever patients, an additional application (the same dose as before) is given if there is still fever within 24 h after the first dose and the interval between two medications ≥ 12 h. The maximum cumulative number is two, and the maximum single dose is up to 800 mg [19].

Finally, an 8-armed study with planned 320 participants will test various combinations of HIV-1 protease inhibitors, oseltamivir, favipiravir, and hydroxychloroquine for the treatment COVID-19 (THDMS-COVID-19) in Bangkok, Thailand. (01.08.2020, https://clinicaltrials.gov/ct2/show/NCT04303299?term=favipiravir&cond=COVID-19&draw=2&rank=3).

There are no current studies listed for sofosbuvir in ClinicalTrials.gov.

Four Trials are listed for oseltamivir, a drug against influenza infection, inhibiting the neuraminidase and thus, the viral spreading after infection of a patient. They do neither eliminate the virus nor are they able to substantially shorten the course of flu disease in patients. Oseltamivir is considered to be helpful in case of a pandemic in order to decrease the viral spread in society. ^[20, 21]^However, an effect against SARS-CoV-2 has never been proven in vitro or in vivo. (https://clinicaltrials.gov/ct2/results?cond=COVID-19&term=oseltamivir&cntry=&state=&city=&dist=&Search=Search)

Ribavirin was tested in vitro 2004 against SARS-CoV and revealed a limited effect on the elimination of SARS-CoV-1; however, the authors concluded that from their pk-data it must be expected that effective concentrations in humans will not be reached by approved human doses [20]. However, ribavirin revealed a higher binding capacity to SARS-CoV-2 RNA Polymerase than remdesivir in recent in silico and in vitro studies [10].

One RCT in Sichuan, China, should be reported evaluating the combination of tenofovir-alafenamide/emtricitabine (TAF/FTC). Although there are no in silico or in vitro data for the binding of TAF to SARS-CoV-2 RNA, the Chinese study plans to evaluate the combination of LPV/r+TAF/FTC vs. LPV/r alone in 60 patients each for the early treatment of COVID-19. Recruiting is still pending due to the International Clinical trials Registry Platform of the WHO (01.08.2020, https://apps.who.int/trialsearch/Trial2.aspx?TrialID=ChiCTR2000029468).

Finally, there are two new studies, already recruiting, listed in the FDA ClinicalTrials.gov database, accessed 10.01.2021: A French trial (AR0-CORONA) evaluates the *Effect of Tenofovir/Emtricitabine Short Course on Viral Clearance in Patients Recently Infected With SARS-COV2 (covid-19) Not Requiring Hospitalization*. The investigators propose a multicenter, open-label, phase 2B/3 randomized trial of a 7-day treatment with TDF/FTC (2 tablets on day 1, then 1 tablet/day for 6 days) according to the dosage used in pre-exposure prophylaxis for HIV. This study is ought to include 60 outpatients (phase 2B) and 120 additional outpatients (phase III) who are diagnosed with SARS-CoV2-positive and have no contraindication to TDF/FTC, without criteria for hospitalization. The primary endpoint of the phase 2B is the SARS-CoV2 antiviral efficacy, quantified by RT-PCR nasopharyngeal sample CT-value increase on day 4 compared to baseline. The primary endpoint of phase 3 will be the rate of non-contagious RT-PCR on day 4 from a nasopharyngeal sample. Secondary endpoints will be tolerance, symptoms resolution, percentage of hospitalization, and the rate of non-contagious RT-PCR on day 7 from a nasopharyngeal sample. This is one of the first studies evaluating an antiviral treatment of SARS-CoV-2 in an outpatient setting, something the ambulatory sector of especially general practitioners are waiting for, since they are currently treating over 90% of all COVID-19 cases.

(10.01.2021, https://www.clinicaltrials.gov/ct2/show/NCT04685512?term=tenofovir&cond=covid-19&draw=2&rank=1)

The second RCT from Colombia refers to the evaluation of the effectiveness and safety of pharmacological therapies used to treat adult patients with COVID-19. Adults aged 18 years or over with a positive RT-PCR or with high suspicion of SARS CoV-2 infection and diagnosis of mild, severe, or critical pneumonia, requiring hospital management at six hospitals in Colombia will be treated with either (1) emtricitabine/tenofovir, (2) colchicine plus rosuvastatin, (3) Emtricitabine/tenofovir plus colchicine plus rosuvastatin, and (4) standard od care (SOC). The study aims to recruit *n*=400 patients in the first step to select the most effective and safe treatments, listed above, and 1200 patients in a second phase evaluating the effectiveness of the selected treatments. The primary outcome is mortality at days 7 and 28. Furthermore, serious adverse events, intensive care unit (ICU) admission, requirement of respiratory support, time to death, number of participants cured, and any adverse event related to treatment will be assessed. Variables for statistical adjustment are sociodemographic and clinical at recruitment, i.e., comorbidities, need for therapeutic support, grade of invasion at admission.

(10.01.2021, https://www.clinicaltrials.gov/ct2/show/NCT04359095?term=tenofovir&cond=covid-19&draw=2&rank=4)

### Side effects/tolerability of nucleosides

In general, the nucleoside analogs can affect the bone marrow and blood cell count, causing hemolytic anemia, decreased hemoglobin and thrombocytopenia. Tenofovir-DF can also decrease bone density and alter kidney function with increased creatinine values. Proteinuria is a symptom of a possible Fanconi-syndrome. Nucleoside analogs also may cause mitochondrial toxicity with peripheral subcutaneous fat loss. However, these side effects usually occur after a long-term intake of NAs, in short periods of treatment such as against SARS-CoV, these side effects only play a minor role. The foremost side effects in the first weeks of treatment are nausea, dizziness, headache, and diarrhea.

### HIV-protease inhibitors

As there is a similarity in the nucleotide sequence of SARS-CoV to SARS-CoV-2 of 79% and to MERS-CoV of 51.8%, and protease is a key enzyme for the replication of coronaviruses, there are data from previous studies in these earlier endemics, which are translated now into therapy concepts for SARS-CoV-2 and COVID-19.

Lopinavir/r was one of four FDA-approved drugs, which were effective against SARS-CoV in cell lines [21], but later investigations revealed contradictory results [3, 5, 22, 23] and the question occurs why this could be. The EC_50_ of Lopinavir was 8μM [21] which is usually reached by BID dosing of Lopinavir/Ritonavir (Kaletra®) 400/100mg in vivo [24], if the drug concentrations in plasma are measured as entire concentration; however, the unbound fraction of Lopinavir is much lower in the plasma due to its protein binding capacity of 98–99%.

Dayer et al. have tested the inhibitory potency of HIV-1 protease inhibitors to coronavirus protease and found the inhibitory potency represented by the similarity of the tested drugs to the certain binding site of Mpro of LPV>RTV>APV>TPV>SQV>ATV>DRV>NFV>IDV, so that in this case, LPV was the most powerful inhibitor of coronavirus protease. The inhibitory potency of Lopinavir in a molecular dynamic simulation was represented by the binding site similarity of 66.67%, whereas the second-generation PI Darunavir/Cobicistat (DRV/COBI) revealed a binding site similarity of 33, 3% [25]. In addition, Nukoolkarn et al. have found in their molecular dynamics simulations, that the active site SARS-CoV 3CL^pro^ is binding to lopinavir/ritonavir through hydrogen bonds [5].

In a retrospective cohort study, *n*=33 adults with laboratory-confirmed COVID-19 without invasive ventilation were given either oral umifenavir (Arbidol®) + LPV/r in the combination group (*n*=16) or oral LPV/r only in the monotherapy group (*n*=17) for 5–21 days. The primary endpoint was a negative conversion rate of coronavirus from the date of COVID-19 diagnosis (day 7, day 14), and it was assessed whether the pneumonia was progressing or improving by chest CT (day 7). Baseline clinical, laboratory, and chest CT characteristics were similar between groups. The SARS-CoV-2 could not be detected for 12 (75%) of 16 patients’ nasopharyngeal specimens in the combination group after 7 days, compared with 6 (35%) of 17 in the monotherapy group (*p* < 0.05). After 14 days, 15 (94%) of 16 and 9 (52.9%) of 17, respectively, SARS-CoV-2 could not be detected (*p* < 0.05). The chest CT scans were improving for 11 (69%) of 16 patients in the combination group after 7 days, compared with 5 (29%) of 17 in the monotherapy group (*p* < 0.05) [26].

In a randomized, open-label trial involving hospitalized adult patients with confirmed SARS-CoV-2 infection, and an oxygen saturation (SaO_2_) of 94% or less, patients were randomly assigned in a 1:1 ratio to receive either LPV/r (LPV/r, 400 mg, and 100 mg, respectively) twice a day for 14 days, in addition to standard care, or standard care alone. The primary endpoint was the time to clinical improvement, defined as the time from randomization to either an improvement of two points on a seven-category ordinal scale or discharge from the hospital, whichever came first. A total of 199 patients with laboratory-confirmed SARS-CoV-2 infection and COVID-19 underwent randomization; 99 were assigned to the LPV/r group, and 100 to the standard-care group. Treatment with LPV/r was not associated with a difference from standard care in the time to clinical improvement (hazard ratio for clinical improvement, 1.24; 95% confidence interval [CI], 0.90 to 1.72). Mortality at 28 days was similar in the LPV/r group and the standard-care group (19.2% vs. 25.0%; difference,−5.8 percentage points; 95% CI, −17.3 to 5.7). The percentages of patients with detectable viral RNA at various time points were similar. In a modified intention-to-treat analysis, LPV/r led to a median time to clinical improvement that was shorter by 1 day than that observed with standard care (hazard ratio, 1.39; 95% CI, 1.00 to 1.91). Gastrointestinal adverse events were more common in the LPV/r group, but serious adverse events were more common in the standard care group. LPV/r treatment was stopped early in 13 patients (13.8%) because of adverse events [27].

LPV/r was also tested in human cell lines and a mouse model against MERS-CoV and revealed lesser therapeutic effects than previously reported. Antiviral activity (EC_50_) in a human lung cell line at concentrations of 8.5μM was low. LPV/r did not stop weight loss or improve hemorrhage, but LPV/r improved pulmonary function in vivo, measured by accessing the FEV1 (Sheahan et al. 2020 Nature).

### Side effects/tolerability of HIV-protease inhibitors

The side effects of HIV-PI are well known and have been extensively studied over the past two decades. The most limiting side effects occur during long-term treatment and include especially lipodystrophy [28] and cardiovascular side effects, which can be life-threatening. But one has to take into account that treatment against COVID-19 will be of very limited duration, so that most probably only short-term side effects will occur, especially headache, dizziness, and diarrhea, well known from the early days of HIV-1 treatment.

### Drug-drug interactions

Poizot-Martin et al. simulated the interaction potential of DAAs with an antiretroviral therapy (ART) in 2500 HIV/HCV co-infected patients of the French Dat´AIDS cohort during the years 2012–2015: 97.1% of patients received a cART with NRTIs, in 43.6% of cases combined with a boosted PI, in 17.3% with NNRTI, 15.4% with INI, and 23.7% of different combinations of these.

The University of Liverpool *Drug Interaction Database* also evaluated contraindications and potential drug-drug interactions between DAAs and antiretrovirals. It was stated that the least contraindications/interactions were found with Sofosbuvir (0.2%/0%) [29, 30].

The interaction potential of ritonavir-boosted HIV-protease inhibitors unfortunately is quite high, so that it is a challenge to combine LPV/r with other currently taken drugs in multimorbid elderly patients, who are the most vulnerable to COVID-19.

### Hepatic metabolism

Cytochrome oxidases (CYP) are involved in human drug metabolism, especially during the intestinal absorption and hepatic first-pass metabolism. Around 60% of all drugs are metabolized via CYP-isoenzymes. Of these ~40% via CYP3A, 25% via CYP2C, 18% via CYP1A, and another 18% via other CYPs. The most important human cytochrome oxidases are CYP1A2, CYP2C19, CYP2C9, CYP2D6, CYP2E1, and CYP3A4. Other important metabolizing hepatic enzymes are transferases, e.g., UDP-glucuronyltransferase (UGT) and *N*-acetyltransferase (NAT).

Regarding the complexity and number of potential DDIs between, especially LPV/RTV and co-medication, the author refers to the package inserts and EMA-INN. Also, the University of Liverpool drug-drug interaction webpage gives a very comprehensive overview (Tables 1, 2, 3 and 4).

**Table 1:** Medication evaluated in clinical trials. Repurposing of HIV protease inhibitors and nucleos(t)ide polymerase inhibitors for the treatment of SARS-CoV-2 infection and COVID-19

**Substance**	**Drug class**	**Status**
Sofosbuvir	Nucleotide polymerase inhibitor	EU-approval 01/2014 against HCV-infection, SOC
Lopinavir/Ritonavir	HIV-protease inhibitor	EU-approval 1999 against HIV-infection, stand-by ART
Remdesivir	Nukleoside analog (polymerase inhibitor)	EU/German approval 04/2020 against COVID-19 in severe cases
Favipiravir	Nukleoside analog (polymerase inhibitor)	Japan - approval against SARS
Tenofovir-Disoproxilfumarate/Emtricitabine	Nukleoside analog (polymerase inhibitor)	EU-approval 2004 against HIV-infection
Ribavirin	Nukleoside analog (polymerase inhibitor)	EU-approval against HCV-infection, out of use

**Table 2:** Past clinical trials

**Substance**	Regimen	**Status**
Remdesivir	RDV, *n*=66, case reports	Compassionate use [13]
Favipiravir	Favipiravir vs. LPV/r, *n*=33	Small RCT [31]
Ribavirin	RBV, *n*=*x*	Case reports in SARS [32–35]
Lopinavir/r	LPV/r, *n*=*x*	Small uncontrolled retrospective trials in SARS-CoV and MERS, case reports in SARS-CoV [26, 34, 36–38]

**Table 3:** A selection of current clinical trials

Substance	Clinical Trial n=	Clinical Trial Name	Participants n=	Comments
Hydroxychloroquine*	183	Solidarity Solidarity Canada Discovery EU Discovery France	775 725 620 160	*majority of trials suspended due to accumulation of adverse events
Lopinavir/r	77	Solidarity Solidarity Canada Discovery EU Discovery France Smaller Trials with combinations of LPV/r and Antibiotics/Antivirals or Antibodies	775 725 620 160	all Mono-arms with LPV/r suspended due to lack of efficacy Dual combinations
Remdesivir	35	Solidarity Solidarity Canada Discovery EU Discovery France NCT 04292899 Expanded Access GS-5734 Expanded Access GS-5734 US-Army MRDC NCT 04292730 ACTT ACTT-II (RDV/Baricitinib) RDV/Tocilizumab	775 725 620 160 6000 ? 262 Study Sites ? 1600 800 1030 225	All RDV Monotherapy Dual combination Dual combination
Chloroquine*	30	Solidarity Solidarity Canada Discovery EU Discovery France	775 725 620 160	*majority of trials suspended due to accumulation of adverse events
Favipiravir	17	FPV/HCl FPV/AZT Further 15 trials	920 660 890	Dual combination with HCl Dual combination with AZT 9 trials are underpowered; mean FPV study arm has n=59
Ribavirin	2	RBV/LPV/r/IF-ß (China) RBV/Nitazoxamide/Ivermectin (Egypt)	50 50	Triple combinations
Tenofovir/FTC	2	AR0-CORONA Colombia	180 100 (phase 2)	Treatment trial in outpatients Treatment trial in inpatients
Sofosbuvir	1	Sofosbuvir or Daclatasvir or Hydroxychloroquine or SOC	25	Underpowered
Total	416 (17.1%)*	Total No. of Participants No. of Participants receiving Remdesivir	16010 (100%) 11935 (74,5%)	

Source: https://www.ncbi.nlm.nih.gov/pmc/articles/PMC7105280/pdf/rpsp-44-e40.pdf

*of all treatment-related studies

Solidarity = Trial/arm stopped; SOC=standard of care; TAF= tenofovir alafenamide; TDF= tenofovir disoproxilfumarate; FTC = emtricitabine; LPV/r = lopinavir/ritonavir; RDV = remdesivir; *Pre-Exposure trials, non-therapeutic

**Table 4:** Combination therapies

**Substance**	**Drug class**	**Probable use**
Remdesivir + LPV/r	NPI + PI	Favorable, scarce data, monitor DDI of LPV/r
Remdesivir + HCLQ	NPI + Antimalarial	Possible, monitor HCLQ side effects
TDF/FTC + LPV/r	NRTI + PI	Possible, moderate side effects, monitor DDI of LPV/r
Favipiravir + LPV/r	NPI + PI	Favorable, scarce but encouraging data, monitor DDI of LPV/r
DRV/COBI + HCLQ	PI + Antimalarial	Unfavorable due to DDI

^a^Source https://ClinicalTrials.gov/

^b^The combination of DRV/COBI and HCLQ is not really reasonable due to the expected DDIs

### P-Glykoprotein (ABCB1)

On their way into target cells, drugs have to overcome different cellular barriers. This can happen by passive diffusion but in many cases also by means of active transport proteins. Most familiar is P-glycoprotein (P-gp), called ABCB1 transporter in new nomenclature, a transmembrane glycoprotein efflux transporter, encoded by the MDR1 gene. This membrane-located ATP-dependent transport mechanism is used by human cells to discharge toxins or drugs identified as toxins.

In humans, P-gp is located in excretory tissues like the intestine, liver, and kidney, but also in the pancreas, heart, and brain or, e.g., tumor cells. The overexpression in tumor cells, e.g., is one reason for the decreased response to cytostatics, known as multidrug resistance. The spectrum of substrates of P-gp is broad and includes drugs, food constitutes, environmental toxins, hormones, amino acids, sugar, or peptides, i.e., molecules from 400 to 2000 Da.

P-gp can be either inhibited or induced, whereas substrates of P-gp often are also substrates of CYP3A4 (Table 5). Obviously, the expression and activity of both are regulated via the same pathways. Thus, the differentiation of effects caused by each is complicated.

**Table 5:** Pharmacological characteristics of currently tested HIV protease inhibitors and nucleos(t)ide polymerase inhibitors drugs against SARS-CoV-2

**Generic name**	**Drug class**	**Molecular weight**	**Plasma protein-binding**	**Metabolism**	**Cellular Transport**	**Additional Information**
Sofosbuvir [39–44]	Nukleoside analog polymerase inhibitor Active metabolite	529.5 g/mol	61–65%	Cathepsin A (CatA), (CES1) und (Hint1). UMP/CMP/NDP- Kinases	P-gp Substrate , BCRP	Prodrug; intracellular phosphorylation to uridintriphosphate-analog
Remdesivir	Nucleotide analog polymerase inhibitor Active metabolite	602.6	n.a.	Phosphorylation	P-gp- and OATP1B1 Substrate	Prodrug; intracellular phosphorylation [45] IC t_1/2_ = 20h
Favipiravir	Nukleoside analog polymerase inhibitor Active metabolite	157.1	n.a.	Phosphorylation via Hypoxanthine-guanine phosphoribosyltransferase P-gp, SLC22- inhibitor	P-gp Substrate+Inhibitor	Prodrug; intracellular [46] phosphorylation to guaninmono/triphosphate-analog
Tenofovir-Disoproxylfumarate	Nucleotide analog polymerase inhibitor Active metabolite	287.2	7.2%	Phosphorylation by Nucleoside Diphosphate Kinase A+B and Adenosinkinase	P-gp Substrate	Prodrug; intracellular phosphorylation to adenosintriphosphate-analog
Ribavirin	Nukleoside analog polymerase inhibitor Active metabolite	244.2	None	Phosphorylation via Adenosinkinase	SLC-Substrate	Prodrug; intracellular phosphorylation to adenosintriphosphate-analog
Lopinavir/r	Proteaseinhibitor (Mpro)	628,8 g/mol	98–99%	CYP3A4 Substrate+Inhibitor (CYP2D6)	P-gp Substrate	Dosing BID
Darunavir/c	Proteaseinhibitor (Mpro)	547.8	95%	CYP3A4 Substrate+Inhibitor	P-gp Substrate	Dosing QD 800 mg or BID 600 mg

**Source**: https://www.drugbank.ca/;*DA* Dalton, ^1^Do not combine with PI^2^ monitor QTc-prolongation; *IC* intracellular, *t*_*1/2*_ half-life, *n.a*. not available, *n.k*. not known

A number of drugs discussed in this editorial are substrates and/or inhibitors of P-gp (Table 5).

## Discussion

The number of ongoing studies regarding the treatment of COVID-19 is impressive:

ClinicalTrials.gov lists 469 studies worldwide (01.10.2020), of which 376 are still active (10.01.2021), but current evidence for the treatment of COVID 19 still is quite low.

There are reasonable arguments from in vitro and drug modeling studies as well as small open trials or case reports supporting the repurposing of already available drugs and possible combinations of such.

However, some turn out to cause more problems in real life than possibly expected. In Sweden, recently all studies with hydroxychloroquine were stopped due to the accumulation of severe side effects in patients (International Press). A pilot study of hydroxychloroquine in the treatment of patients with common coronavirus disease-19 revealed expected side effects: Four cases (26.7%) of the HCQ group and 3 cases (20%) of the control group had transient diarrhea and abnormal liver function (*p*>0.05). The authors conclude that the prognosis of common COVID-19 patients is good. However, the results are neither valid nor very encouraging for hydroxychloroquine [47].

A 200-mg oral dose of hydroxychloroquine has a half-life of 537 h or 22.4 days in the blood, and 2963 h or 123.5 days in the plasma. Patients experiencing an overdose may present with headache, drowsiness, visual disturbances, cardiovascular collapse, convulsions, hypokalemia, rhythm, and conduction disorders including QT prolongation, torsade de pointes, ventricular tachycardia, and ventricular fibrillation [48, 49] (FDA-Approved Drug Products: Hydroxychloroquine Oral Tablets). This may progress to sudden respiratory and cardiac arrest. Another concern is irreversible retina damage, when taken over a longer period. Although these side effects are rare, the result can be dramatic. [50] The use of the anti-malarial drug chloroquine to treat COVID-19 patients has recently been halted at several Swedish hospitals due to reported side effects such as cramps and loss of peripheral vision. (01.07.2020, https://www.webmd.com/lung/news/20200407/side-effects-halt-use-of-chloroquine-vs-COVID-19).

Remdesivir seems to be the most promising candidate for the treatment of SARS-CoV-2 infection and COVID-19, but as 68.5% of all studies investigate Remdesivir + X, this could cause problems if the drug reveals either less efficacy than expected or side effects which were not obvious in the past in the very limited number of patients treated because of Ebola infection. In this case, trials and alternative treatments with other comparators would be missing. In addition, remdesivir is produced by one single manufacturer, so that production capacities’ supply chains may not match with the worldwide requirement of COVID-19 treatment, the more as the USA already bought 500.000 doses of remdesivir in July, which represents the entire worldwide production July-September 2020.

In addition, the results of the WHO guided Solidarity trial, recently published in *BMJ*, could not reproduce these findings, as neither overall mortality nor severe courses of COVID-19 were decreased in patients receiving remdesivir (*n*=2570). The WHO *Living Guidelines* now refrains from recommending remdesivir as the primary treatment of COVID-19 in severely ill patients and states that further studies should be conducted regarding this issue [17].

Although a number of case reports from Europe and China are showing heterogeneous results regarding the outcome of HIV-1 and SARS-CoV-2 co-infected patients [51–57], a final conclusion that HIV-protease inhibitors and nucleoside reverse transcriptase inhibitors are inefficient in the treatment of COVID-189 cannot be drawn, as the limitations of these reports are quite clear: The reported outcomes refer to smaller, retrospective, and uncontrolled case series of only symptomatic patients. Asymptomatic cases may have been missed and data were partially incomplete, such as information about the onset, duration, and intensity of symptoms [53]; the plasma concentrations of TDF; or HIV-protease inhibitors, which might influence the efficacy of treatment against COVID-19. Furthermore, bacterial coinfections which were reported from a study in the USA [56] were a major reason for mortality in hospitalized patients with HIV and COVID-19. One study even reported a smaller proportion of patients with HIV and COVID-19 compared to the entire hospitalized population due to COVID-19 [57].

However, the data from NEAT indicate *n*=903 cases of reported HIV-1 and SARS-CoV-2 co-infection in Europe until January 25, 2021, with a high rate of hospitalizations (*n*=374) and deaths (*n*=68, https://neat-id.knack.com/neat-id-COVID-19#home/). Taken the number of 85279 patients being treated in the reporting sites with an incidence of 1.06%, this corresponds to an incidence of 0.024% in the entire population of people living with HIV and AIDs in Europe (https://www.who.int/hiv/data/en/), the incidence of SARS-CoV-2-positive tested subjects in Europe in the entire population is 0.4%. (https://de.statista.com/), and the prevalence of PLWH in Europe ranges from 0.1% (e.g., Germany) up to 1.2% (Russia) (https://cfs.hivci.org/). Although reports in the NEAT database may be missing and we actually do not know the true rate of infections in people living with or without HIV and AIDS, the difference seems to be significant. There have been also reports from South Africa that PLWH taking tenofovir as part of their cART vs. those not taking tenofovir do have a lower incidence of mortality during a COVID-9 infection. This remained apparent even after adjusting for confounders such as renal disease, viral suppression, and ART duration, although the overall incidence in PLWH for COVID-19 mortality was higher than in the uninfected population (adjusted hazard ratio [aHR] 2.14; 95% confidence interval [CI] 1.70-2.70) [58].

### Outlook

In summary, it becomes quite clear that (1) the majority of all discussed trials are conducted as monotherapy studies, partially vs. placebo. (2) Only a small number of trials are evaluating dual combination regimens, in doing so testing also unusual or even contraindicated combinations (e.g., Darunavir/Cobicistat/Hydroxychloroquin). (3) Currently, no triple/multiple therapy regimens are evaluated in RTCs. (4) Ribavirin, the nucleoside analog with the highest binding capacity to SARS-CoV-2 RNA, is only part of two small trials. (5) Tenofovir/DF/AF, a nucleotide analog, which is available worldwide at a reasonable price as a generic drug, is evaluated in only two currently recruiting RCTs as part of COVID-19 therapy. (6) Lopinavir/ritonavir is only tested as monotherapy in seriously ill COVID-19 patients and in this case did not reveal sufficient efficacy [59], as already seen in the past [27, 60]; Lopinavir/ritonavir is not evaluated as part of a combination therapy [26].

Alternative approaches and up-to-date study designs which deploy the currently available medication in reasonable ways are scarce.

(A) There are no head-to-head studies of promising candidates, such as remdesivir vs. RBV vs. TDF/FTC vs. FPV + X. TDF shows structural similarity to remdesivir, its binding energy to RNA polymerase is little less [10] and it is available worldwide as a generic drug, storage, and supply doesn’t need cooling, application orally QD. The binding capacity of RBV is even higher than that of RDV and favipiravir already showed a comparable efficacy in smaller pilot studies.
(B) There are no double or triple combination studies, as established in the treatment of HBV, HCV, or HIV, e.g., RDV + X or TDF/FTC + X or TDF/FTC+LPV/r or Favipiravir + X, LPV/r + X.
(C) There is only one study evaluating the repositioning of tenofovir in an outpatient setting as early antiviral treatment against SARS-CoV-2. More studies for the ambulant antiviral treatment are warranted as the capacities of hospital care are exhausted worldwide.

The repurposing or repositioning of drugs for the new indication of SARS-CoV-2 and COVID-19 treatment is more than reasonable. It can be the fastest way to treatment of COVID-19 and the embankment of the pandemic. But this approach needs international concerted policies regarding science, production, supply, and access of/to drugs. These are federal responsibilities, questions of independent science and marketing independent supply, and finally the general access to the best available treatment.

Studies should be conducted coordinated and have comparable study designs to allow meta-analyses. Smaller trials can provide a basis for the generation of new hypotheses or therapeutic approaches and should be very carefully appraised regarding their necessity, ethical, and scientific concerns. All of these cannot be committed to either the manufacturer’s side or single academic research units. The final aim must be finding the most effective therapy in the shortest time, assuring worldwide access to treatment, independent from production capacities or market power.

*RDV* Remdesivir *LPV/r* Lopinavir/Ritonavir, *RBV* Ribavirin; *RCT* randomized controlled trial

Trial/arm stopped, *SOC* standard of care, *TAF* tenofovir alafenamide, *TDF* tenofovir disoproxilfumarate, *FTC* emtricitabine, *LPV/r* lopinavir/ritonavir, *RDV* remdesivir, *Pre-Exposure trials non-therapeutic

*TDF* tenofovir disoproxilfumarate, *FTC* emtricitabine, *LPV/r* lopinavir/ritonavir, *RDV* remdesivir, *HCLQ* hydroxychloroquine, *NPI* Nucleos(t)ide polymerase inhibitor (SARS-CoV-2), *NRTI* nucleos(t)ide reverse transcriptase inhibitor (HIV-1), *PI* protease inhibitor (SARS-CoV-2 Mpro or HIV protease), *DRV* darunavir, *COBI* cobicistat, *DDI* drug-drug-interaction
